# Leveraging collaborative research networks against antimicrobial resistance in Asia

**DOI:** 10.3389/fpubh.2023.1191036

**Published:** 2023-12-07

**Authors:** Shiying He, Pami Shrestha, Adam Douglas Henry, Helena Legido-Quigley

**Affiliations:** ^1^Saw Swee Hock School of Public Health, National University of Singapore, Tahir Foundation Building, Singapore, Singapore; ^2^School of Government & Public Policy, University of Arizona, Tucson, AZ, United States; ^3^London School of Hygiene and Tropical Medicine, London, United Kingdom

**Keywords:** antimicrobial resistance, social network analysis, health policy, international cooperation, Asia, One Health, collaboration in AMR, barriers in AMR research

## Abstract

**Background:**

Antimicrobial resistance (AMR) is a global health security threat requiring research collaboration globally and regionally. Despite repeated calls for international research collaboration in Asia, literature analyzing the nature of collaborative AMR research in Asia has been sparse. This study aims to describe the characteristics of the AMR research network in Asia and investigate the factors influencing collaborative tie formation between organizations.

**Methods:**

We carried out a mixed-methods study by combining social network analysis (SNA) and in-depth interviews. SNA was first conducted on primary data to describe the characteristics of the AMR research network in Asia. Exponential random graph models (ERGMs) were then used to examine the influence of factors such as organization type, country affluence levels, regional proximity and One Health research on collaborative tie formation among organizations. In-depth interviews were conducted with network participants to provide contextual insights to the quantitative data.

**Results:**

The results reveal that the research network exhibits a core-periphery structure, where a minority of organizations have a significantly higher number of collaborations with others. The most influential organizations in the network are academic institutions from high-income countries within and outside Asia. The ERGM results demonstrate that organizations prefer to collaborate with others of similar organization types, country-based affluence levels and One Health domains of focus, but also with others across different World Health Organization regions. The qualitative analysis identified three main themes: the challenges that impede collaboration, the central role of academic institutions, and the nature of collaborations across One Health domains, giving rise to important empirical milestones in understanding AMR research in Asia.

**Conclusion:**

We thus recommend leveraging academic institutions as “integrators” to bridge differences, increasing funds channelled towards research capacity building to alleviate structural barriers to collaboration, streamlining collaborative mechanisms to overcome cumbersome administrative hurdles, and increasing efforts to establish trust between all organizations.

## 1 Introduction

Antimicrobial resistance (AMR) is a phenomenon where microbes such as bacteria, viruses, and fungi develop resistance to modern medicine designed to treat the infectious diseases they cause (1). The role of antimicrobials such as antibiotics and antifungals are crucial not just in treating infections, but also in protecting patients from fatal infections after medical procedures such as surgery and chemotherapy (2). AMR caused 700,000 deaths in 2014 (3), with significant increase to 1.27 million deaths in 2019 globally (4). The persistent overuse and misuse of antimicrobials across human medicine and food production sectors has been a major driver of AMR emergence (4), and with insufficient new drugs in the pipeline to replace ineffective ones, the AMR threat is widespread and serious. Conservative estimates point towards an astounding 10 million additional deaths annually by 2050 and a cost of at least US$100 trillion if no action were taken to contain it (3, 5). Experts have also felt that the AMR agenda risked losing momentum even prior to the COVID-19 pandemic, with the pandemic’s increased use of antimicrobials threatening further acceleration of AMR emergence (6).

This global health security threat looms much more heavily over the Asia Pacific region than in other regions of the world (1, 7). The region is home to the World Health Organization (WHO) Southeast Asian region (SEAR), which has been identified as a major hotspot for AMR emergence and transmission and is expected to see antibiotic consumption by humans and animals jump exponentially by 2030 (1, 8). Given that the Asia Pacific is home to two-thirds of the world’s population, ten of the world’s least developed countries, rapidly growing farming sectors, burgeoning populations and overall weaker health system infrastructures, the region is likely to see a compounding of the potentially devastating effects of AMR (1).

The AMR issue has been considered a “wicked problem” given its involvement with many stakeholders with competing interests and complex interactions (9). Stakeholders with vested interests in the AMR issue comprise a diversity of sectors and interests- including the public spheres of policymaking and governance, as well as the private and for-profit pharmaceutical and agricultural industries. Adding to the complexity of formulating effective and sustainable policies is the need for joint policy responses across the interconnected spheres of human, animal and environmental health, making a One Health approach essential.

Research collaboration is critical to combating this wicked AMR problem. However, literature analyzing the nature of the collaborative AMR research network in Asia has been sparse. This paper thus sets out to first describe the characteristics of the AMR research network in Asia; and second, to investigate the factors that influence the formation of collaborative ties between organizations within the AMR research network. The AMR research network refers to the patterns of collaboration among organizations that are collectively engaged in advancing knowledge about AMR.

WHO Global Action Plan (GAP) on AMR launched in 2015 included targeted research as their second strategic objective (10). As the importance of international collaboration in managing the AMR problem is widely recognized, international health organizations, national leaders, as well as academics have actively pushed for the creation of international collaborative networks.

Studying of AMR and international collaborative research networks is important for several reasons, including:

1. Allowing for the pooling of resources and expertise and acting as a “force multiplier” in the advancement of scientific research (11, 12).
2. Aiding the harmonization of research activities which can promote best practices and the investigation of issues relating to the transnational nature of AMR within regions (13).
3. Resolving current misalignments in research activities and ensuring that their relevance to the region’s true priorities and knowledge gaps (11, 14).

Evaluating the structure of collaborative AMR research networks in Asia is a critical need amidst increased research funding but decreasing political momentum on the AMR issue (15). The increased attention on AMR globally has not translated into necessary policy actions and ground-up initiatives. Moreover, despite repeated calls for collaboration between academics in Asia, academic work that analyzes the region’s existing AMR research networks has been consistently absent (16, 17). The existing literature is limited to several studies on the scale and scope of AMR research in other regions (13, 16–20), as well as bibliometric analyzes examining the disciplinary characteristics of AMR-related publications in the past decade (21, 22). In Asia, while there have been reports examining regional AMR policy and surveillance networks, there is little academic work that specifically analyzes collaborative relationships between nations or institutions in the region (23, 24).

In sum, there is a lack of literature detailing the current state of AMR research collaboration in the region despite their importance in driving more effective resource mobilisation to combat AMR. Hence, to advance the containment of AMR in the region, there is a need to produce a structured and comprehensive layout of the AMR research network in Asia. We thus carried out a mixed-methods study by combining the use of social social network analysis (SNA) and in-depth interviews.

## 2 Materials and methods

This is a mixed-method study that combined SNA and in-depth interviews to achieve two objectives. The first objective is to describe the network of AMR research collaborations in Asia and identify the factors that influence the formation of these collaborative ties between organizations within the network. The second objective is to understand the opportunities and barriers to AMR research collaborations in Asia. A mixed-method approach allows for the quantitative description of the AMR research network and the factors that are associated with collaboration, while providing context behind the observed structure of the network. In this paper, we use the term “network” to refer to the myriad collaborations that occur between various types of organizations. Network “structure” refers to the configuration of these collaborations among organizations. The term “organization” refers to distinct members of the network; in this study, they are defined as entities, which can include but are not limited to the following: academic institutions, non-governmental organizations (NGOs), international organizations and policy makers.

### 2.1 Learning, network segregation, and homophily

*Learning* refers to the collective ability of participants within a network to produce knowledge about shared problems and deploy this knowledge to successfully manage these problems (25, 26). An *integrated* network promotes effective research because collaborations between otherwise divided organizations are more likely to enable the coupling of diverse sets of knowledge systems, skills, and resources that exist throughout the network, while *segregated* networks (the opposite) tend to inhibit these outcomes (27). Although the importance of integration in networks is widely understood as a critical driver of learning, networks typically tend towards segregation— where network participants tend to be connected to others similar to themselves. A major driver of network segregation is *homophily,* which is the tendency for individuals to form ties based on their similarity to others (28, 29).

In AMR research networks, homophily may drive segregation in several traits including organization type, affluence, regional proximity, and One Health domain focus. Firstly, *organization types* can be understood as different categories of AMR research stakeholders. Examples include universities, research consortia, hospitals, government agencies, industry, non-governmental organizations (NGOs) and international organizations (IOs), among others. Studies have found that barriers such as differences in institutional norms, research priorities, strategic orientation and potential conflicts of interest are known to impede collaboration (30). Secondly, *affluence* refers to the amount of resources that a country is likely to channel towards research activities. Studies have also found that organizations based in affluent countries are home to expertise and funding that promote collaboration, and simultaneously attract scientists with substantial scientific knowledge, skills and social connections who are often from equally affluent countries (31–34). Thirdly, *regional proximity* refers to the geographical distance between researchers. Most researchers tend to work with professionals that fall within their professional circles, oftentimes within the same institutional setting, with distance and language differences found to be barriers to collaboration (35). Lastly, One Health domain focus refers to a specialization in one or more of the human, animal or environmental health domains within The One Health approach. This approach investigates health issues through the examination of interactions and interdependencies between the three domains (36) and studies have found the persistent neglect of the animal and environmental health domains in AMR (37–39).

Homophily in any of these traits impedes learning and collective research of the AMR problem. Thus, we investigate whether homophily in these four factors influence the formation of collaborative ties between organizations within the AMR research network. The nature of our research design lends itself to the following four hypotheses.

*Hypothesis 1*: Organizations tend to form ties on the basis of similarity in organizational type.

*Hypothesis 2*: Organizations tend to form ties on the basis of similarity in affluence level.

*Hypothesis 3*: Organizations tend to form ties on the basis of regional similarity.

*Hypothesis 4*: Organizations tend to form ties on the basis of One Health domain similarity.

This study departs from previous works by utilizing a mixed-methods approach that leverages the joint advantages of quantitative methods to elucidate the AMR research network and the factors that influence collaborative tie formation, as well as the qualitative methods in providing observations about the context within which a network lies, or in other words, what is “going on” within a network (40).

### 2.2 Social network analysis

This study uses SNA to describe and model the system of AMR research collaborations in Asia. SNA is a widely-used set of methodological tools to analyze the social relationships between individuals, groups of individuals or organizations (41, 42). It is useful in understanding both the nature of ties that connect participants and identify the most influential participants within a system (43, 44).

### 2.3 Participants

This study began by defining the boundaries of the AMR research network to be captured by using a combination of positional and relational strategies. A positional strategy refers to the inclusion of participants within a network based on “actor attributes, membership in an organization or having a well-defined position for inclusion in a network” and can be understood as a form of purposive sampling (42). A relational strategy engages “knowledgeable informants or the network actors themselves to nominate additional actors for inclusion” and is akin to snowball sampling (42). This combined strategy aimed to capture the network as comprehensively as possible.

Research participants were recruited via purposive sampling followed by snowball sampling based on the following recruitment criteria. First, the participant had to either be from a research institution based in Asia or be collaborating with a research institution based in Asia. As described in an earlier section, participants could represent a variety of organizations, including but not limited to academic institutions, NGOs, international organizations and ministries. Second, the participant had to be involved in an aspect of AMR research, including but not limited to AMR surveillance, stewardship, public education, infection prevention and control or research and development. An initial seed list of AMR researchers was purposefully compiled to kickstart the snowballing process, which involved the selection of authors from two landmark documents and the research team’s personal contacts. The first report was a landmark journal article in the Lancet’s 2015 *Antimicrobials: access and sustainable effectiveness* series (45). 10 out of 22 of the report’s authors were included in the seed list based on the study’s recruitment criteria. The second document was a report summarising the national AMR efforts of each ASEAN member state to establish a strategy for collaboration within the region (14). All 26 names in the document were included in the seed list. The authors of the journal article would represent a global health security perspective on AMR, while the report would account for a local perspective on the AMR threat in the region. The final step in compiling the seed list involved identifying participants from China, Hong Kong, Japan, South Korea, and Taiwan, which were countries yet to be represented in the list, through personal contacts.

### 2.4 SNA data

All individuals were invited to participate in the study via emails obtained from public domains. During the survey, participants were asked to nominate any number of key individuals and organizations involved in AMR research in the region they have collaborated with. This study defined “collaboration” broadly to mean “being involved in a joint research effort wherein involvement includes the discussion of ideas, sharing of data, reviewing each other’s papers, exchange of physical materials and co-authorship” (see Supplementary Appendix 1).The term “collaboration” is used to describe relationships between individuals, between organizations, and between individuals and organizations (31). A broader conceptual definition of “collaboration” beyond mere co-authorship of journal articles was adopted to produce a more concrete network map unconfined by resources that facilitate co-authorship. The term “organizations” was also clarified for participants to include “schools, research institutions, non-governmental organizations, international organizations, think tanks, policy-makers, etc.” All nominees were subsequently contacted, and the snowballing process continued until saturation was reached. A maximum of three emails were sent before a participant was marked as a non-respondent. Nominees whose contact information were not publicly available were not contacted. Recruitment started in July 2020 and ended in September 2021 with a response rate of 22.3%. This meant that out of 184 nominees invited, 41 had participated in the email survey.

### 2.5 SNA analysis

After the data collection phase, data was cleaned and aggregated at the organizational level on Excel (46). This was performed to capture network characteristics at a more macroscopic level (35), where collaborations between nominated organizations and organizations that participants were primarily affiliated with rather than that between individuals. Tools of SNA were then deployed to map and describe the characteristics of the AMR research network. From this point onwards, the term “network participants” and “organizations” will be used interchangeably.

To test our four hypotheses, exponential random graph models (ERGMs) were used. ERGMs model the probability of observing a network mapped from a dataset amidst a series of randomly generated graphs (47, 48). It is a statistical tool used to simulate the preferences for specific network configurations of network participants that would result in the formation of the observed network structure (47, 49). These preferences for specific network configurations will be used as independent variables, which then represent the configuration of ties that are observed in the network. All models presented in this paper are convergent, which means that simulations that use the obtained coefficients will result in a distribution of graphs where the resultant network is the representative or average graph derived from all independent variables in the model (48). The ERGMs used to test all four hypotheses included the following network configurations:

- *Organization similarity.* This variable captured a tendency of organizations to form ties with other organizations that belong to a similar organization type. Organizations were categorised based on the following types: academic institution, research network or professional organization, hospital, government entity, NGO, industry, media, funding organization or IO. Organizations that share at least one type in common are defined to be similar, while those sharing none at all are defined to be different.
- *Regional similarity.* This variable captured a tendency of organizations to form ties with other organizations that belong to countries in closer geographical proximity. Regional distance was captured based on WHO regions (50–52). Organizations which share at least one region in common are defined to be similar, while those which share no regions at all are defined to be different.
- *Affluence distance.* This variable captured a tendency of organizations to form ties with other organizations that belong to a country with a similar level of affluence. We capture the distance in affluence levels between the countries each organization belongs to by first generating a composite *Affluence* variable comprising a country’s income group, human development index (HDI) and GDP *per capita* (50, 53, 54). *Affluence distance* then takes the log of the absolute value of the difference between each pair of organization’s affluence levels based on the *Affluence* variable. The larger the difference between the level of affluence of countries between each pair of organizations, the larger the affluence distance.
- *One Health domain similarity.* This variable captures a tendency of organizations to form ties with other organizations working on the same One Health domain. An organization’s involvement in a particular One Health domain is an aggregation of all the domains its affiliated individuals are involved in. Data for this variable had only been collected from participants who responded to the survey. Organizations which share at least one domain in common are defined to be similar, while those which share none at all are defined to be different.
  Since it was not possible to pinpoint the country of origin or main site of operation for regional and global organizations, the ERGMs were run on national organizations only.

### 2.6 In-depth interviews

A second set of data collected to understand the processes and context behind tie formation were the use of in-depth interviews. 15 participants were purposively selected from the seed list, which had been compiled based on our recruitment criteria. All participants held senior positions at relevant organizations and had substantial experience in AMR research in the region. The sample spanned 10 countries, including Cambodia, China, Hong Kong, Indonesia, Japan, Malaysia, Pakistan, Philippines, Singapore and Thailand. Supplementing our quantitative data using qualitative data could provide valuable insights into the detailed context behind collaborative tie formation, which cannot always be derived from quantitative data.

### 2.7 Interview data

In-depth interviews that spanned approximately 30 min to 1 h were conducted by one of the authors between July 2020 and December 2021. All interviews were carried out in English over Zoom or Skype. In view of the limited number of researchers involved in international collaborations in the region, data collection ceased once thematic saturation was attained. Participants were asked to reflect on the nature of their collaborations with others on AMR in the region, how collaborations were formed and the barriers that impede them, if any. The detailed topic guide for the interview is provided in Supplementary Appendix 2.

### 2.8 Interview analysis

All interviews were audio-recorded, transcribed verbatim, verified by the interviewer and analyzed in QRS NVivo (55). Considering the potentially sensitive nature of the interviews in relation to policy-making processes, all transcripts were fully anonymized. Thematic analysis was conducted to elicit new themes on the insights relevant to the processes of collaborative tie formation among organizations in the network. Thematic analysis was conducted in four steps: Familiarization of data; Identification of codes and themes; Line by line coding; Organizing codes and themes. Full transcripts were re-reviewed fully to re-contextualize all coded data. We then summarized the data based on the themes that emerged and ceased data collection once thematic saturation was reached. All data were then summarized based on the themes that emerged.

### 2.9 Ethical considerations

This study received an exemption for ethical approval (institution anonymized). All participants in the quantitative portion of the study were informed that participation conveyed implicit consent, and those in the qualitative portion provided verbal consent at the beginning of the interview.

## 3 Results

### 3.1 Quantitative findings

**Figure 1:**
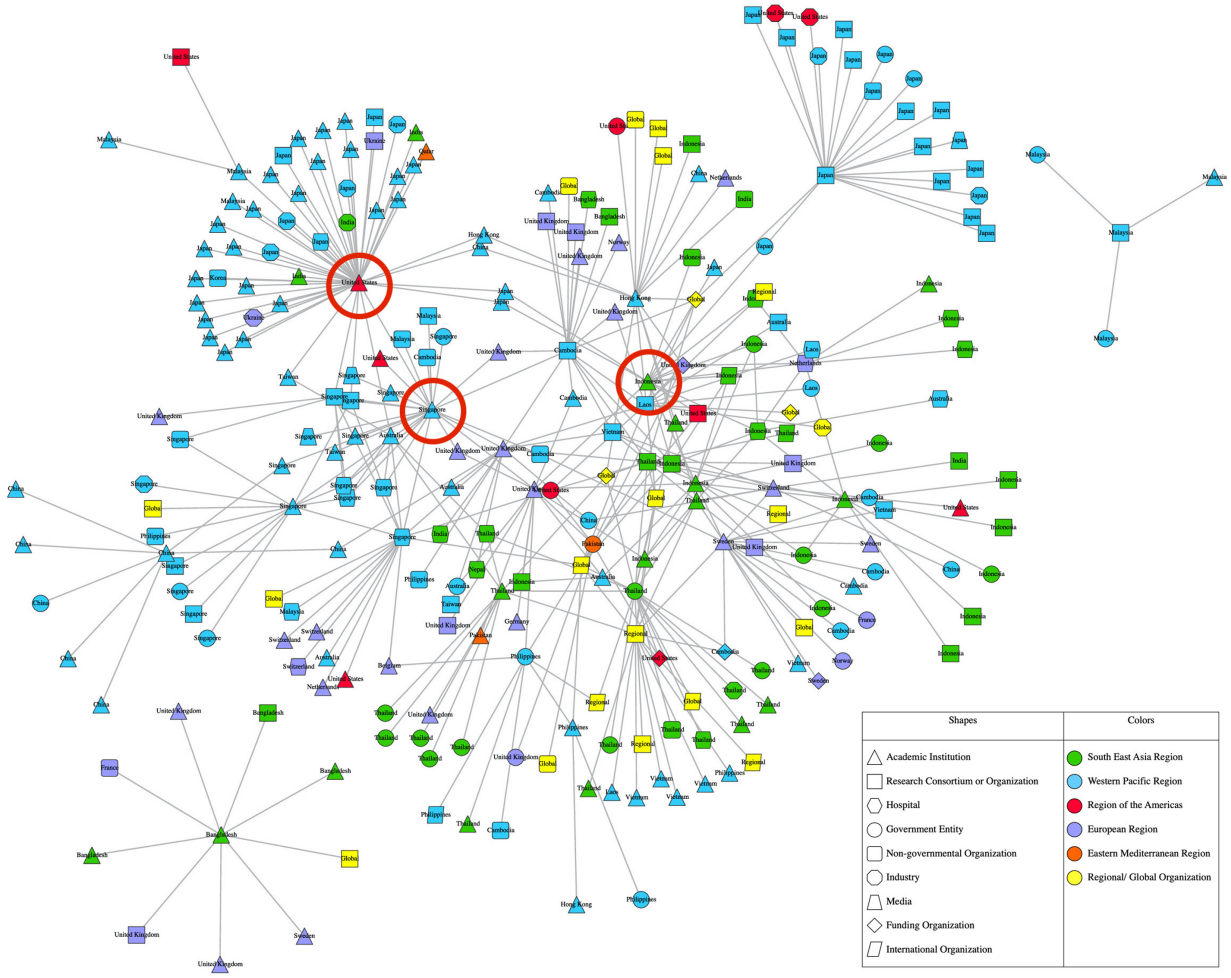
Full AMR research network in Asia.

**Figure 2:**
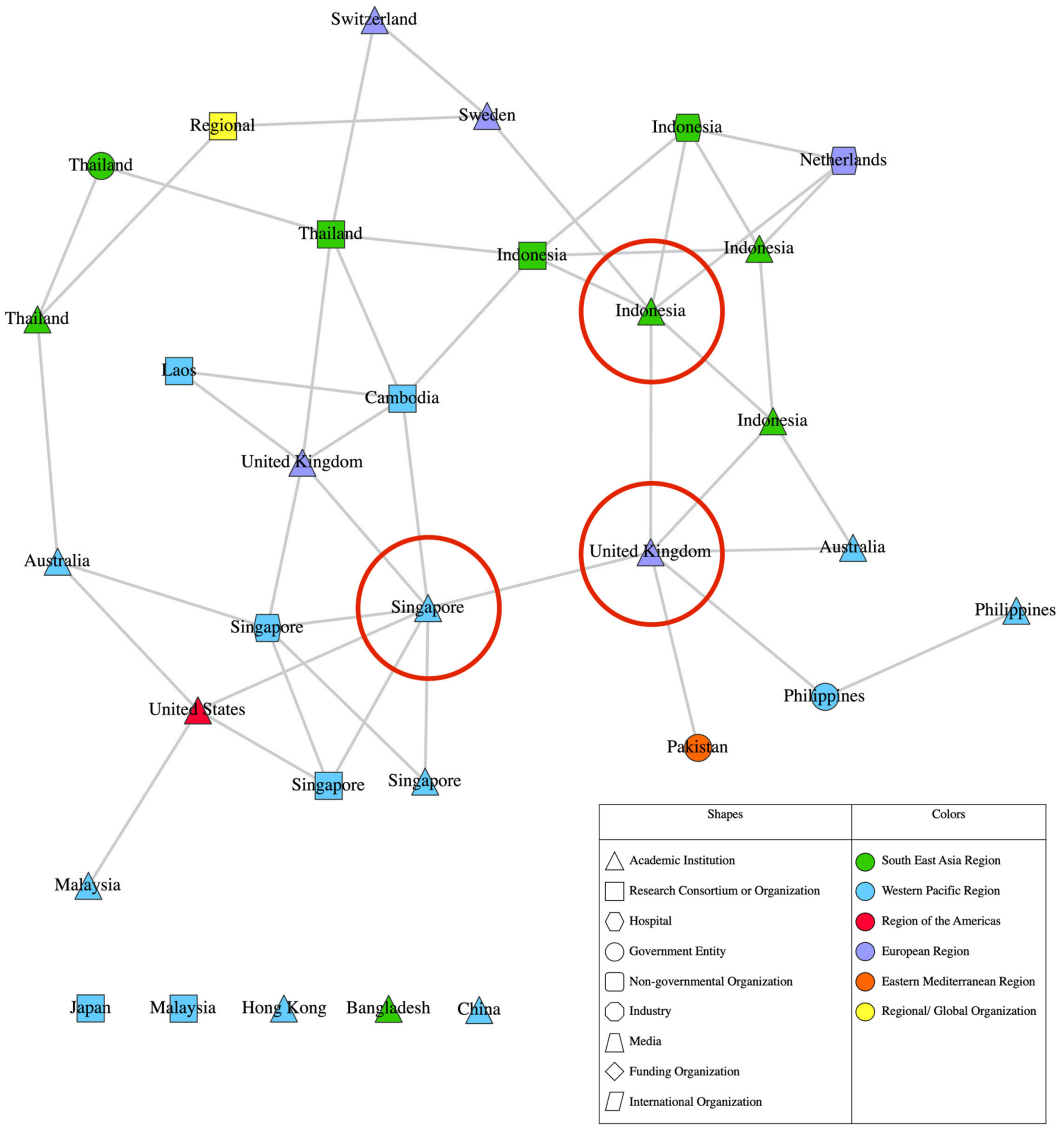
Reduced AMR research network in Asia.

This study has constructed and analyzed two networks from all data collected, depicted in Figures 1, 2. Figure 1 is a “full network,” which includes all network participants. Figure 2 is a “reduced network” which only includes survey participants. While Figure 1 (full network) depicts the broad array of organizations, both core and peripheral, Figure 2 (reduced network) provides a zoomed-in view of the core of the network. The ERGM results show that organizations in the AMR network preferred to collaborate with others of similar organization types, affluence levels and those working on similar One Health domains. The results also reveal that organizations prefer to collaborate with others from different WHO regions. The ERGM results are reported in full below and in Table 1.

**Table 1:** Exponential random graph model results.

	Full network	Reduced network
Organizational similarity	0.572^***^ (0.0827)	0.421 (0.252)
Affluence distance	−0.230^***^ (0.0128)	−0.324^***^ (0.0397)
Regional similarity	−0.541^***^ (0.115)	−0.326 (0.304)
One Health domain similarity	–	0.634^**^ (0.318)
Number of organizations	266	32
Number of collaborative ties	723	103

Results show the log odds-ratios of each factor. ERGMs were run conditional on the number of edges in each network, which means that all randomly generated graphs would have the same number of ties as our observed networks. The standard error of parameter estimates are indicated in parentheses. Asterisks indicate the significance of parameter estimates. ^*^p < 0.05; ^**^p < 0.01; ^***^p < 0.001.

In Figures 1, 2, nodes represent each unique organization that had participated or been nominated in the study. All nodes are connected by edges which represent collaborative ties between individuals from each organization, aggregated at the organization level. Nodes are labelled based on their country of origin or main site of operation, and the shape of nodes represents their primary organization type. Nodes are colored according to the WHO region they belong to. The top three most central organizations from each network are denoted by a red circle. All data analysis was carried out in R (56) using the hRU R package (57). All network visualizations were created with Visone (58).

Figure 1 reveals that the full network is large, with a total of 266 nodes. It is also diverse, with organizations originating from different countries and constituting a variety of organization types. Collaboration occurred within and outside organizations’ own countries and regions, and across different organization types. Most nodes from the full network have between a total of 1 to 3 collaborative ties with other nodes, while a minority of nodes have 20 or more ties with other nodes. Similarly, most of the nodes from the reduced network have between 1 to 2 ties with other nodes, while a minority of nodes have 6 or more ties with other nodes. This distribution of ties tells us that the AMR research network exhibits a core-periphery structure, where a minority of organizations hold a monopoly over collaborative ties with others and are more well connected compared to the majority of organizations in the network.

This study uses one specific concept of centrality called betweenness centrality, which measures the extent a network participants placed in a position that grants it access to the shortest paths between other pairs of participants in the network. The higher an organization’s betweenness centrality value, the more other organizations depend on it to connect with other organizations and are possibly the best positioned to facilitate collaboration between other organizations. Notably, two of the top three most central organizations of both networks are from high-income countries (all three are denoted by a red circle in Figures 1, 2). The most central organization in the full network is an academic institution from the United States, followed closely by one from Singapore, then another from Indonesia. In the reduced network, the most central organization is the *same* academic institution from Singapore, followed closely by an academic institution from the United Kingdom that was in the fourth position in the full network, with the third-most central organization being the *same* academic institution from Indonesia. The overlap in the most central organizations across both networks demonstrates the influence of these academic institutions from Singapore and Indonesia.

Both figures seem to reveal regional segregation, particularly between the Southeast Asia Region (SEAR) and Western Pacific Region (WPR). The green nodes across both figures, show that there appears to be a distinct collaborative “cluster” of organizations within the SEAR. A similar pattern can be seen among blue nodes, which point to another collaborative “cluster” of organizations within the WPR. Meanwhile, organizations from other WHO regions such as the European Region (EUR), Region of the Americas and the Eastern Mediterranean Region appear to be interspersed throughout both networks. The top three most central organizations also belong to three different regions- WPR, SEAR and the Region of the Americas from the full network; and WPR, EUR and SEAR in the reduced network.

**Figure 3:**
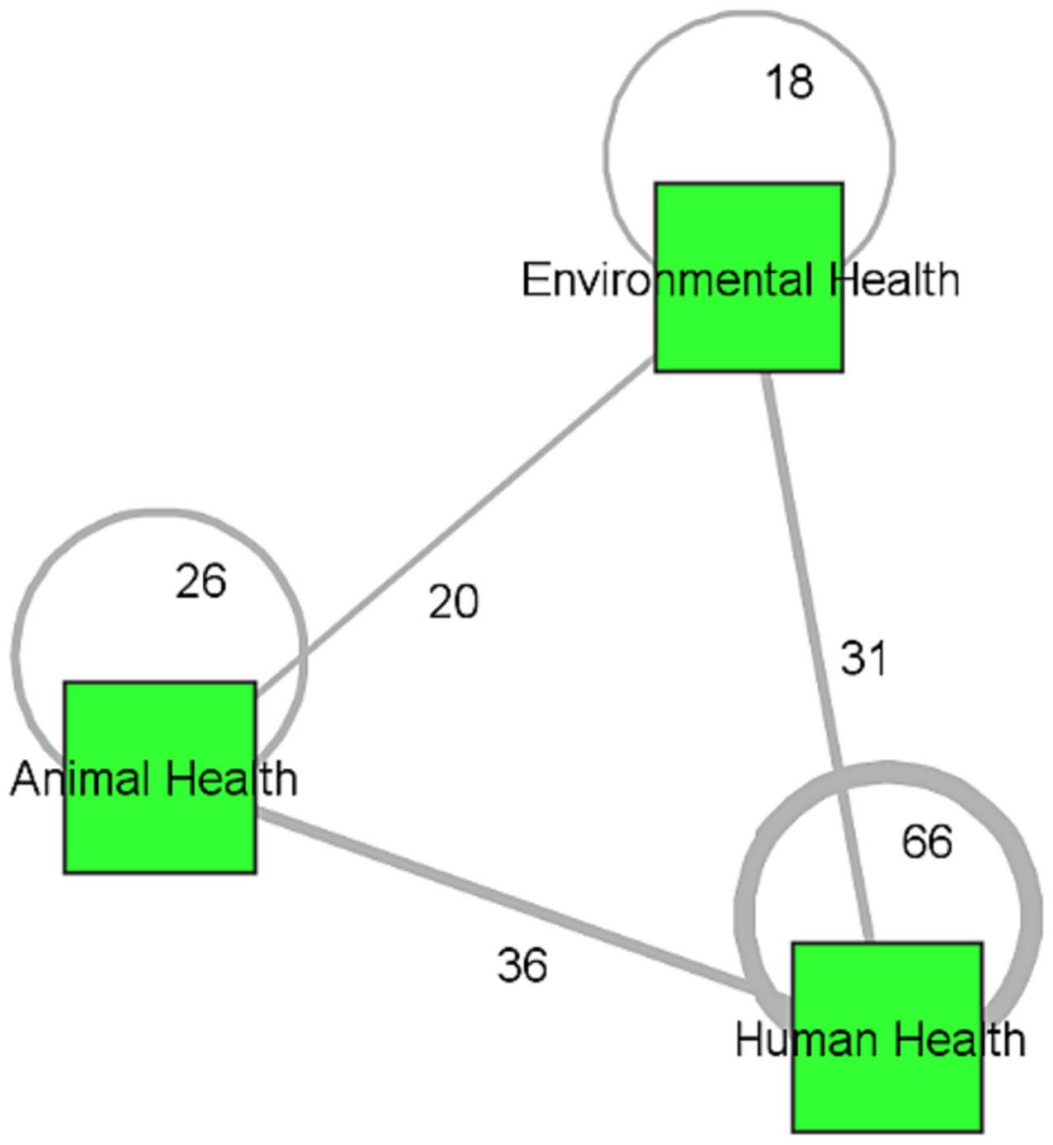
One Health domain attention network.

From our survey respondents only, we were able to directly collect data on the One Health domains they focus on. Figure 3 depicts the frequency with which organizations associated with a particular domain collaborate with others associated with the same or different domain. The graphic shows that most collaborative ties are among organizations that *both* participate in human health (66 collaborations), and the most frequent collaborations *across* domains are between human and animal health (36 collaborations) and between human and environmental health (31 collaborations). Overall, there appears to be a healthy amount of integration between domains, though the extent of collaboration between environment and animal domains is relatively less frequent.

Table 1 summarises the ERGM results for both the full and reduced networks. Firstly, organizational similarity is positive in both networks, but only significant in the full network. Consistent with Hypothesis 1, organizations are more likely to form ties with other organizations that have at least one organization type in common. For example, academic institutions are more likely to collaborate with other academic institutions or hospitals that are affiliated with academic institutions compared to collaborating with an NGO or IO. Secondly, affluence distance is negative and significant in both the full network and reduced network. Consistent with Hypothesis 2, the larger the difference in affluence level between organizations, the less likely they are to collaborate. In other words, organizations are more likely to collaborate with others of similar affluence levels. Thirdly, regional similarity is negative in both networks, but only significant in the full network. This means that contrary to Hypothesis 3, organizations are not confined to working with others within their own regions, but instead prefer to expand their networks and collaborate with organizations that belong outside their WHO region. Finally, One Health domain similarity is positive and significant in both the full network and reduced network. This means that consistent with Hypothesis 4, organizations are more likely to collaborate with one another if they have at least One Health domain in common.

### 3.2 Qualitative findings

We present our findings from the in-depth interviews under three main themes. The data suggests that the AMR research network presented several barriers to collaboration, identified the central role of academic institutions as well as present collaborations across One Health domains.

#### 3.2.1 Challenges that impede collaboration

Participants reported that organizations across countries face multiple challenges attempting to form collaborative ties. This barrier was raised particularly for those in less affluent countries. A participant had observed a prevailing preference for siloed work that had been reinforced by a lack of institutional mechanisms to facilitate collaboration. Other participants cited familiar challenges, particularly cumbersome administrative and funding hurdles that impede collaboration.

I think the most pressing, and I’ll talk in a bigger perspective for the developing country, is the lack of the surveillance system. Because if you do not know the exact position of anything in the country, you would not be able to move ahead (in collaboration) (Participant 8).

Most of the people have been trained in their discipline. They are thinking of silo. I mean, so it’s a bit difficult to think out of the box when you are having your own life trained to be a good data (manager), a good medical doctor or agronomist. And after even for the new generation, the mechanisms of collaboration are not even there. When (collaborations) are there, they are not functioning because there is blocking process funding but not only about administrative process way of how to make uh people from different agency or different department being able to run something together or who is going to lead when you have three different ministry and department, all that. So all that it’s what,… How will people collaborate? (Participant 12).

It was reported that collaboration was also impeded by differences in level of expertise as well as the extent of communication and trust between organizations. The next few quotes illustrate the varying degrees of trust and communication within organizations’ collaborative activities.

They have some training but we do not know what the extent of that training is in terms of antimicrobial resistance and how do they know which, what is effective, what is appropriate practice or how do they keep up to date with information, if at all. So my sense is that there’s this kind of discord… between what happen on the ground and what happens in the policy level, which I think has not really been addressed, because a lot of the policy guidelines that are put out are not necessarily things that are very applicable in some of these on the ground (Participant 2).

Building trust is very difficult…….academic institutions can work on certain things but I think, like I said before, there’s just some sensitivities around certain subject matter (Participant 13).

I think the AMR community in Singapore is quite small. So there are periodically calls for funding. There are conferences or local symposiums. So I think you get to form the networks that way. And also because it’s more or less quite a collegial group, so people pull other people in (Participant 15).

#### 3.2.2 Central role of academic institutions

Academic institutions were named the centrepieces in driving AMR research collaborations. The following quote illustrates the prominence of the “publish or perish” phenomenon present in academia that drives both the research agenda as well as the collaborations that follow:

I think, you know, for us we still rely on the publication model because that’s what’s built into the institution for promotion in ten years, so I think there’s (academic) institutional drivers that are dictating that that’s what people should do… (Participant 13).

Moreover, academic institutions are regarded not only as technical experts, but also as neutral parties at AMR-related discussions. The next quote shows how their positions are associated with objectivity that may be critical to the policy research and decision-making process in AMR:

If I’m invited as a moderator, by which I have been for the past five, six years, sometimes a few times a year for AMR, I will always say yes just because being in the university, if you have the that moderating role, the way that you ask questions I think is different compared to if you have somebody from the ministry itself to moderate. Just because being in the academy, the way you ask questions can be very neutral (Participant 5).

Participants had revealed that universities within and between countries also tended to be better connected to one another.

Southeast Asia and where we have a lot of different collaboration…it’s Thailand, Laos, Cambodia, Vietnam, Indonesia and Philippines uh with different international organization, NGOs, um uh research institute…. In Thailand with X University. And after we are working in Laos it’s Y University. In Phnom Penh it’s Z University. In Vietnam we have several universities (Participant 12).

#### 3.2.3 Collaborations across One Health domains

The findings show an encouraging acceptance of the One Health approach among participants. The following quotes highlight familiarity with the approach, as well as the presence of collaborative work on two or more One Health domains:

Oh mine is more One Health. So understanding the how antibiotic has been used and then understanding how to say very basic epidemiological type of research but trying to include the One Health aspect from the animal health to environment to the potential impact on human health issues. So looking across the various elements of One Health from animal interface, environmental interface into the human is what I do (Participant 5).

We use One Health approach and we surveyed and the behaviour of human that use antibiotic. But that human is human who have dogs and cats and kind of. And then and also veterinarian and veterinary assistant that working together with dog and cats. Yeah. Look at their behaviour for prescription or consume antibiotic. And we try to find the linkage between of resistant pattern between animals and human as well (Participant 7).

Understandably, collaborations in human health continue to take priority, in so doing neglecting other domains that receive insufficient public funding, as illustrated in the following quote:

For those countries there’s a lot of emphasis on… human health they think about public health. When we think about public health, a lot of… funding will go to the Ministry of Health. But the veterinary services by which is really important also for public health, if any of the if any countries, any of these countries were to suffer any kinds of uh budget cuts, veterinary service would be the first one to go, basically. To be cut in terms of their funding. They do not think about veterinary health as or veterinary services as a really major component of public health (Participant 5).

However, there remains a higher tendency for collaboration within similar disciplines. This tendency is driven by the ease of navigating familiar professional norms as seen in this quote:

People carry their education and professional culture habits with them, which means they will be prone to collaborate (with similar researchers), you do not want to collaborate…with people beyond your own world, your comfort zone… (Participant 11).

## 4 Discussion

### 4.1 Key findings

Our mixed-methods study combined SNA and in-depth interviews to elucidate the AMR research network in Asia and the factors that influence collaborative tie formation. The quantitative findings reveal that the network exhibits a core-periphery structure, where a minority of organizations are more well-connected and have a significantly higher number of ties with other organizations, while most organizations are only connected to one to two other organizations. The most influential organizations are academic institutions from HICs within and outside Asia. Among One Health domains, most collaborations occur among organizations in human health. ERGM results show that organizations within the AMR research network preferred to collaborate with others of similar organization types, affluence levels and One Health domains, but also with others from different WHO regions. Our qualitative data extend our quantitative findings by providing the context behind tie formation. The qualitative analysis found that the AMR network had existing barriers to collaboration, academic institutions had central roles in driving collaboration, and revealed the presence and extent of collaborations across One Health domains.

There are three main ways the qualitative results supplement the results of the quantitative findings. First, the interview results helped pinpoint the ways the most central organizations could control communications and facilitate collaborations. The quantitative findings illustrated the network’s core-periphery structure and that the most influential organizations were primarily academic institutions from both within and outside Asia. Prior research ascertains that public health remains one of the few fields where cross-stakeholder collaboration is more common (59). While other organizations such as IOs, NGOs and government entities have been identified in this network, academic institutions remain the most central in driving collaborations because of their role as drivers of the research agenda, neutral moderator of discussions between stakeholders and promoter of international collaborations with other academic institutions.

Second, it illuminated the structural factors that impede collaboration, which enabled us our understanding on the processes by which collaborative ties form between organizations. Affluence appears to be a key factor. This was seen from how two of the top three most central organizations from both networks were from HICs. The quantitative findings also revealed the presence of network segregation on the basis of affluence level. Based on the qualitative analysis, less affluent countries may lack the requisite AMR data and infrastructure to participate in collaborative initiatives. This finding is consistent with other studies, where affluence and access to resources are required to both initiate and maintain collaborative ties (34). The quantitative analysis also found the presence of segregation based on organizational similarity. The qualitative results points to bureaucratic, administrative and funding barriers, as well as a lack of trust and AMR-related expertise that impede collaboration between different organizations. This finding is also consistent with other studies, wherein collaborations between academia and government associations were difficult when researchers and policymakers have different priorities, or when policymakers lack expertise or belief in evidence-based policymaking (59).

Third, the qualitative results indicate potential areas for future research. For instance, a notable finding from the quantitative analysis was the preference of organizations to collaborate with others from a different WHO region. Current studies suggest otherwise, since language barriers and cultural differences serve as barriers to communication (35, 60). A possible explanation for this finding could be that organizations based in the West serve as significant sources of funding and expertise for organizations in the region (61). Similarly, the quantitative analysis found that organizations were more likely to collaborate with others working on similar One Health domains. The qualitative analysis revealed nuances behind this preference, showing that even amidst a professional tendency to collaborate with others from a similar domain, there is a move towards embracing the One Health approach.

### 4.2 Policy recommendations

Our team thus puts forth the following recommendations. Engaging all stakeholders is key to truly multi-sectoral AMR research collaboration. A Comprehensive Review of the WHO GAP AMR performed in 2022 identified a need for greater coordination among international and national partners in order to facilitate the implementation of the WHO GAP (62). In view of academic institutions as “integrators”, or the most central organizations in the network, they can be tapped on as a resource to facilitate the initiation of new collaborative avenues, mediators in connecting previously segregated organizations as well as sources of information to be harnessed for best practices in encouraging collaboration (63). For instance, they could be tasked to leverage their collaborative ties and organize the necessary conferences and other networking events for all stakeholders to promote the familiarization of one another’s institutional norms (33).

Next, considering the barriers posed by affluence levels, sources of funding should not only be directed towards carrying out research work, but also towards health systems strengthening and AMR research capacity building. It has been found that only one in five countries were able to find funding sources to support full implementation of their national action plans (62). Initiatives that alleviate structural barriers are therefore required as far as possible to engage network participants equitably regardless of affluence background, particularly given the transnational nature of the AMR problem. Moreover, given the significant challenges stemming from organizational differences, there is a need for high-level stakeholders to invest resources to understand the resources invested in collaborations within and across organizations, and streamline these collaborative mechanisms (64). This will help all organizations overcome the high costs of navigating differences in institutional norms and funding structures. A study on the challenges and opportunities to equitable AMR research collaborations has also proposed a complementary streamlining of research funding mechanisms to enable a more flexible allocation of resources to projects that span different stakeholders across countries and regions (61).

The unique nature of research collaboration, which leverages personal networks, institutional connections and academic ties, enables it to bypass political boundaries and root the seeds for future political collaborations (33). Efforts are also required to establish and build trust between all organizations, where differences are perceived not as weaknesses and impediments but as strengths that can be leveraged upon in collaboration, and trust sustained through the attainment of mutually-beneficial goals (65, 66). Other successful attempts at promoting cross-sectoral collaboration required the establishment of specific governance structures, such as strategic advisory committees that are representative of expertise from all relevant scientific domains, as well as a leadership group to steer research coordination and project management (67). Research collaborations across organization types, country affluence levels, region and One Health domain involvement should thus be highly encouraged to promote regional momentum on fighting AMR at both the academic and political levels.

By employing a mixed methods design, this study has examined both quantitative and qualitative data to provide a more holistic understanding of the characteristics of the AMR research network in Asia. This study is the first of its kind to systematically address gaps in information about the nature of AMR research collaboration in the region, as well as the first to have collected and analyzed primary data to understand the factors that influence the formation of collaborative ties. This paper thus provides an important evidence base for funders, policymakers, academics and other stakeholders to develop future roadmaps, initiatives, and structures to enhance collaboration between organizations within the AMR research network in the region to further the AMR agenda.

### 4.3 Study limitations

However, there are some limitations of our study. First, we had a relatively typical response rate of 22.3% as found in other network and policy social network studies. This could be because its duration coincided with the beginning of the COVID-19 pandemic in 2020. Participants in our survey may hence be more likely to be involved in AMR research, which the team has tried to minimize by reaching out to each survey nominee a total of three times over the course of the study before classifying them as a non-respondent. Second, our study is also limited by a possible sampling bias. Due to resource constraints, our seed list was limited to two key papers on AMR and the team’s personal contacts. Doing so may have excluded other key network participants within the network. The team has hence tried to minimize sampling bias by selecting two papers that discussed AMR at the global and regional levels.

## 5 Conclusion

Along with increased political, academic and private investment in AMR research, it is ever more imperative to have a strategic, cohesive direction for AMR research in the region. Duplication and inefficient coordination can be costly- especially when it comes to combating an insidious silent pandemic such as AMR. In a region with mixed needs, research collaboration is ever more important. Our study provides critical empirical data that describes the characteristics AMR research network in the region, identifies the most central organizations and identified factors that influence collaborative tie formation. Our study thus offers insights into more focused investments in strengthening AMR research networks in the region, which ultimately increases the odds of producing more creative and novel research that can drive the needle forward in combating AMR.

## Data availability statement

The raw data supporting the conclusions of this article will be made available by the authors, without undue reservation.

## Ethics statement

This study received an exemption for ethical approval from the National University Singapore (NUS) Institutional Review Board (IRB) for Social, Behavioural, and Educational Research (SBER). The participants provided their written informed consent to participate in this study.

## Author contributions

SH: conceptualization, methodology, software, formal analysis, investigation, data curation, writing – original draft, writing – review and editing, and visualization. PS: investigation, data curation, writing – original draft, writing – review and editing, and project administration. ADH: methodology, software, formal analysis, resources, data curation, writing – original draft, writing – review and editing, visualization, and supervision. HL-Q: conceptualization, methodology, investigation, writing – review and editing, supervision, and funding acquisition. All authors contributed to the article and approved the submitted version.

## Glossary

AMR: antimicrobial resistance
ASEAN: Association of Southeast Asian Nations
CoSTAR-HS: The Collaborative Solutions Targeting Antimicrobial Resistance Threats in the Health System
COVID-19: Coronavirus Disease of 2019
ERGMs: exponential random graph models
EUR: European Region
GAP: Global Action Plan
HICs: high-income countries
hRU R: Henry R Utilities
IOs: international organizations
IRB-SBER: Institutional Review Board – Social, Behavioural and Educational Research studies
NGOs: non-governmental Organizations
NUS: National University Singapore
QRS NVivo: Qualitative Research Software Developer NVivo
SNA: social networks analysis
SEAR: South-East Asia Region
WHO: World Health Organization
WPR: Western Pacific Region
