# Supplementary material for: Leveraging collaborative research networks against antimicrobial resistance in Asia
Source: Front Public Health. 2023 Dec 7;11:1191036. doi: 10.3389/fpubh.2023.1191036 (PMC10749297; doi:10.3389/fpubh.2023.1191036)
Supplement: Supplementary file 1 [file Table_1.DOCX]

Supplementary Material

**Title: Leveraging Collaborative Research Networks against Antimicrobial Resistance in Asia: A Mixed-Methods Analysis**

**Shiying He, Pami Shrestha, Adam Douglas Henry^*^, and Helena Legido-Quigley Correspondence:** Adam Douglas Henry adhenry@arizona.edu

# Supplementary Data

**Supplementary Appendix 1: SNA Survey Questionnaire**

1. Which One Health domain do your research interests lie? You can select more than one option.

a. Human health

b. Animal health

c. Environmental health

For the following questions, please list as many answers as you can.

2. Please identify organizations that you have collaborated with within the course of your work on the AMR research, in your capacity as a representative of your organization. (‘*Organisations’ can include schools, research institutions, non-governmental organisations, international organisations, think tanks, policy-makers etc.
‘Collaboration’ here refer to* being involved in a joint research effort wherein involvement includes the discussion of ideas, sharing of data, reviewing each other's papers, exchange of physical materials and co-authorship.*)*

a. Who are the people you work with specifically in the organizations that you mentioned?

3. Which organizations do you wish to work with and why?

4. Please identify organizations and people that you believe have the most influence on AMR research in Asia.

5. Which researchers would you recommend we speak to seek their views on AMR, both in your country and in the region? You are welcome to mention as many people as you think would be useful.

**Supplementary Appendix 2: Question Guide for In-Depth Interviews**

Question Guide for In-Depth Interviews

Please elaborate on your role on AMR researchers.

1. How is the issue of AMR framed in your particular research setting? (Is AMR framed as a “healthcare”, “innovation”, “development”, “security”, “One Health” issue?)
2. What research areas in AMR do you think should be prioritised in Asia right now?
3. What do you think about the current state of collaboration between AMR researchers in the region now? Do you see any patterns of collaboration?
4. Which institutions or organizations are leading the collaborative AMR research in the region?
5. What are the barriers the researchers or organizations are facing while collaborating or initiating a collaborative AMR research? Please elaborate.
6. How do you exchange research information with your research collaborators? Do you see a need for improvement for such information exchange, if so, how?
7. How do you think collaborative AMR research can be fostered in the region?
